# Ketamine: A tale of two enantiomers

**DOI:** 10.1177/0269881120959644

**Published:** 2020-11-06

**Authors:** Luke A Jelen, Allan H Young, James M Stone

**Affiliations:** 1 Department of Psychological Medicine, Institute of Psychiatry, Psychology and Neuroscience, King’s College London, London, United Kingdom; 2 South London and Maudsley NHS Foundation Trust, London, United Kingdom

**Keywords:** Ketamine, (S)-ketamine, (R)-ketamine, depression, NMDA receptor, AMPA receptor, BDNF, TrkB, mTORC1, ERK, GSK-3, 5-HT, dopamine, opioid receptor

## Abstract

The discovery of the rapid antidepressant effects of the dissociative anaesthetic ketamine, an uncompetitive N-Methyl-D-Aspartate receptor antagonist, is arguably the most important breakthrough in depression research in the last 50 years. Ketamine remains an off-label treatment for treatment-resistant depression with factors that limit widespread use including its dissociative effects and abuse potential. Ketamine is a racemic mixture, composed of equal amounts of (S)-ketamine and (R)-ketamine. An (S)-ketamine nasal spray has been developed and approved for use in treatment-resistant depression in the United States and Europe; however, some concerns regarding efficacy and side effects remain. Although (R)-ketamine is a less potent N-Methyl-D-Aspartate receptor antagonist than (S)-ketamine, increasing preclinical evidence suggests (R)-ketamine may have more potent and longer lasting antidepressant effects than (S)-ketamine, alongside fewer side effects. Furthermore, a recent pilot trial of (R)-ketamine has demonstrated rapid-acting and sustained antidepressant effects in individuals with treatment-resistant depression. Research is ongoing to determine the specific cellular and molecular mechanisms underlying the antidepressant actions of ketamine and its component enantiomers in an effort to develop future rapid-acting antidepressants that lack undesirable effects. Here, we briefly review findings regarding the antidepressant effects of ketamine and its enantiomers before considering underlying mechanisms including N-Methyl-D-Aspartate receptor antagonism, γ-aminobutyric acid-ergic interneuron inhibition, α-amino-3-hydroxy-5-methyl-4-isoxazolepropionic receptor activation, brain-derived neurotrophic factor and tropomyosin kinase B signalling, mammalian target of rapamycin complex 1 and extracellular signal-regulated kinase signalling, inhibition of glycogen synthase kinase-3 and inhibition of lateral habenula bursting, alongside potential roles of the monoaminergic and opioid receptor systems.

## Introduction

There are significant limitations to current widely prescribed antidepressant treatments. These include a significant delay in the onset of therapeutic action (weeks to months) and approximately one-third of patients with major depressive disorder (MDD) failing to demonstrate an adequate response (Al-Harbi, 2012). For individuals with depression, particularly if suffering from suicidal ideation, these time lags and resistance to standard treatments can be extremely harmful (Hantouche et al., 2010).

Increasing evidence has revealed that the dissociative anaesthetic ketamine, an uncompetitive N-Methyl-D-Aspartate (NMDA) receptor antagonist, has the potential to overcome such limitations, demonstrating rapid antidepressant and anti-suicidal effects, even in treatment-resistant patients (Coyle and Laws, 2015; Kishimoto et al., 2016). It has been proposed that ketamine’s antidepressant effects are primarily mediated through NMDA receptor antagonism, resulting in disinhibition of pyramidal cells and an acute cortical glutamate surge, with downstream effects on synaptogenesis and neuroplastic pathways (Lener et al., 2017). However, the precise molecular and cellular processes underlying ketamine’s antidepressant effects are still not clear and evidence suggests that mechanisms other than NMDA receptor inhibition play a more crucial role in the antidepressant effects of ketamine, its component enantiomers and metabolites (Jelen et al., 2018; Zanos et al., 2016).

In this review, we summarise findings on the antidepressant effects of ketamine and its enantiomers. We then discuss underlying therapeutic mechanisms, exploring the case that ketamine’s enantiomers and metabolites may produce complementary antidepressant effects via distinct mechanisms, before considering future directions of enquiry.

## Ketamine enantiomers and metabolites

**Figure 1.**
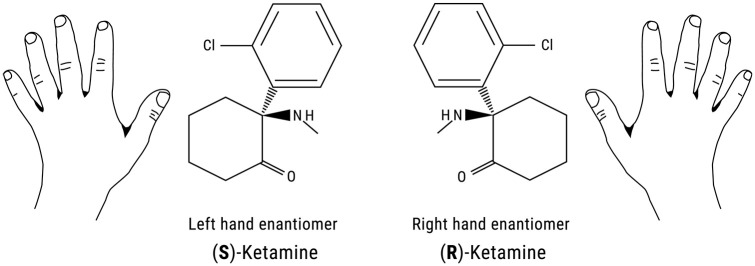
Chemical structure of ketamine enantiomers. (S)-ketamine and (R)-ketamine are a pair of stereoisomers that are non-superimposable mirror images of each other. An example of familiar objects that are related in such a way are the left and right hand.

**Figure 2.**
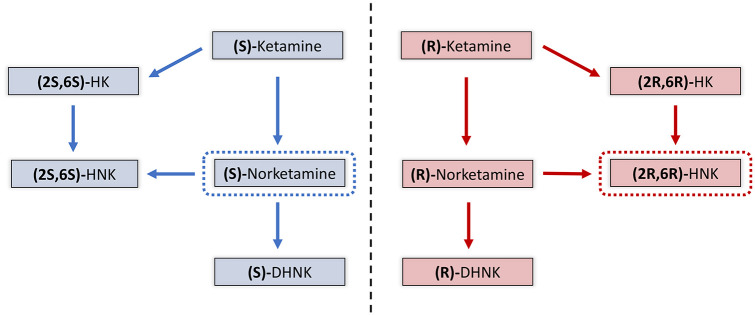
Major metabolites of (S)-ketamine and (R)-ketamine. (S)-ketamine or (R)-ketamine are initially metabolised to (S)-norketamine or (R)-norketamine via CYP3A4 or CYP2B6. (S)-norketamine or (R)-norketamine are further metabolised to (S)-dehydronorketamine (DHNK) or (R)-DHNK. (S)-norketamine or (R)-norketamine are metabolised to (2S,6S)-hydroxynorketamine (HNK) or (2R,6R)-HNK via CYP2A6. (S)-ketamine or (R)-ketamine may also be metabolised to (2S,6S)-HK or (2R,6R)-hydroxyketamine (HK) via CYP2A6 before transformation to (2S,6S)-HNK or (2R,6R)-HNK. Metabolites identified as candidate antidepressants are highlighted in dashed boxes.

Ketamine is a racemic mixture that consists of equal amounts of two enantiomers, (S)-ketamine and (R)-ketamine (or esketamine and arketamine) (Figure 1). (S)-ketamine has a three to fourfold greater binding affinity for the NMDA receptor than (R)-ketamine (Ki = 0.30 μM and Ki = 1.4 μM respectively) (Ebert et al., 1997). In humans, (S)-ketamine is more potent than (R)-ketamine both as an anaesthetic and as an analgesic, which is putatively explained by its higher affinity for the NMDA receptor (White et al., 1980, 1985). It was argued that because of its increased potency, lower doses of (S)-ketamine could be used in anaesthesia/analgesia with faster recovery times and therefore potentially some diminution in dissociative and psychotomimetic side effects (Kohrs and Durieux, 1998). However, direct comparative studies of (S)- and (R)-ketamine have suggested otherwise. In one study, higher rates of psychotomimetic side effects were seen in an (S)-ketamine treated group, despite the dose of (S)-ketamine being lower than (R)-ketamine (0.45 mg/kg and 1.8 mg/kg respectively) (Mathisen et al., 1995). Furthermore, a healthy volunteer study from Vollenweider et al. (1997) found that although (S)-ketamine administration produced acute psychosis-like reactions (ego-dissolution, illusions and hallucinations, thought disorders, paranoid ideations), in the same individuals (R)-ketamine did not produce any psychotic symptoms but instead led to a state of relaxation and a feeling of wellbeing.

(S)-ketamine and (R)-ketamine both undergo extensive metabolism by cytochrome P450 enzymes to corresponding forms of norketamine, dehydronorketamine (DHNK), hydroxyketamine (HK) and hydroxynorketamines (HNKs) (Zanos et al., 2018; Zarate et al., 2012a) (Figure 2). (S)-ketamine or (R)-ketamine is first demethylated by either CYP3A4 or CYP2B6 to (S)-norketamine or (R)-norketamine. (S)-norketamine or (R)-norketamine are subsequently metabolised to (S)-DHNK or (R)-DHNK or HNKs. Hydroxylation of (S)-norketamine or (R)-norketamine by CYP2A6 at the six position results in (2S,6S)-HNK and (2R,6R)-HNK respectively, which are the major HNK metabolites found in plasma following ketamine infusion (Moaddel et al., 2010; Zarate et al., 2012a). CYP2A6 can also directly hydroxylate (S)-ketamine or (R)-ketamine to form (2S,6S)-HK and (2R,6R)-HK, which are further transformed to (2S,6S)-HNK or (2R,6R)-HNK (Desta et al., 2012). Of these metabolites, (2R,6R)-HNK and (S)-norketamine have attracted particular interest as candidate antidepressants in their own right (Yang et al., 2018a; Zanos et al., 2016).

## Ketamine as an antidepressant

**(R,S)-ketamine:** In the first double-blind, placebo-controlled study of racemic ketamine in MDD, it was demonstrated that a single sub-anaesthetic intravenous (IV) infusion (0.5 mg/kg over 40 mins) resulted in rapid antidepressant effects, within hours of administration (Berman et al., 2000). A number of subsequent studies have also demonstrated the rapid-acting antidepressant effects of ketamine in treatment-resistant unipolar and bipolar depression (Price et al., 2009; Zarate et al., 2006, 2012b). Findings have been reviewed in several meta-analyses, which report robust antidepressant and anti-suicidal effects, lasting up to 1 week, in treatment-resistant MDD and bipolar depression (Kishimoto et al., 2016; Wilkinson et al., 2018), with acute dissociative symptoms being the most commonly reported side effect (Kishimoto et al., 2016).

Unfortunately, the antidepressant effect of a single dose of ketamine is not generally sustained beyond 1 week (Kishimoto et al., 2016). In the first randomised controlled trial (RCT) of repeated ketamine administration, it was shown that twice- or thrice-weekly administration of IV ketamine (0.5 mg/kg over 40 mins) was sufficient to maintain antidepressant efficacy over 15 days in individuals with treatment-resistant depression (TRD) (Singh et al., 2016b). Similar findings have also been reported in open-label repeated infusion studies (Rasmussen et al., 2013; Shiroma et al., 2014; Zheng et al., 2018). In contrast, a recent RCT investigating the effects of six ketamine infusions (0.5 mg/kg over 45 mins) or saline placebo over 3 weeks in severe TRD with current, chronic suicidal ideation failed to demonstrate a significant difference in depression severity or suicidality at the 3 week endpoint (Ionescu et al., 2019). However, this study was limited by a small sample size (out of 26 randomised patients, *n*=13 per group, only 14 completed the entire study) and therefore may have been underpowered to detect a true difference between treatment groups. In addition, all patients were maintained on their medication regimes throughout the infusion phase and the impact that concomitant medications may have had on ketamine’s effects cannot be ruled out.

Although there are specialist centres around the world and an increasing number of ketamine clinics in the United States offering ketamine infusions for depression (Ketamine-Clinics-Directory, 2020), the use of repeated infusions may not be the most practical due to the resources required. Other routes of administration (oral, sublingual, intranasal, intramuscular or subcutaneous) could prove to be simpler and more feasible alternatives for repeated administration but few studies have evaluated these options (Andrade, 2017).

**(S)-ketamine:** As NMDA receptor antagonism was understood to play a key role in ketamine’s antidepressant mechanism, (S)-ketamine was investigated as a novel antidepressant candidate by Janssen Research & Development due to its higher affinity for the NMDA receptor. In a first proof-of-concept trial, IV (S)-ketamine at doses of 0.2 mg/kg and 0.4 mg/kg led to rapid and robust antidepressant effects in individuals with TRD (Singh et al., 2016a). Side effects included headache, nausea and dissociation. It was suggested that as improvements in depressive symptoms were not significantly different between the two tested doses that a lower dose of (S)-ketamine may allow for better tolerability while maintaining efficacy (Singh et al., 2016a).

A fixed-dose (S)-ketamine nasal spray has subsequently been developed and tested in TRD. A number of Phase II and III trials have shown that intranasal (S)-ketamine plus an existing or newly initiated oral antidepressant outperforms placebo plus an oral antidepressant for individuals with TRD (Canuso et al., 2018; Daly et al., 2018, 2019; Popova et al., 2019), although others failed to demonstrate positive results (Fedgchin et al., 2019; Ochs-Ross et al., 2020). In a large discontinuation study, 297 individuals with TRD who met response or remission criteria following 16 weeks of treatment with intranasal (S)-ketamine (56 mg or 84 mg twice weekly) plus an oral antidepressant were entered into a randomised withdrawal phase (to continue with (S)-ketamine or switch to placebo) (Daly et al., 2019). Those randomised to continue treatment with intermittently administered (S)-ketamine nasal spray plus an oral antidepressant had a significantly delayed time to relapse compared to those treated with placebo nasal spray and oral antidepressants. A subsequent open-label study has examined the long-term safety of (S)-ketamine nasal spray plus a new oral antidepressant in patients with TRD (Wajs et al., 2020). Common treatment-emergent adverse events included dizziness, dissociation, nausea and headache, which mostly occurred on dosing days, were mild to moderate in severity and resolved on the same day. Longitudinal analysis showed dissociative symptoms declined over subsequent administrations and cognitive performance was generally found to either improve or remained stable compared with baseline. Similar long-term maintenance or safety evidence of this level are not available for (R,S)- or (R)-ketamine at this time.

Considering the available evidence, the United States Food and Drug Administration and European Medicines Agency have approved the (S)-ketamine nasal spray, *Spravato*^TM^, for adults with TRD in combination with an oral antidepressant. However, some questions remain regarding uncertainty of efficacy, safety, potential for abuse and need for careful monitoring, which currently limit wider use (Kryst et al., 2020; Turner, 2019).

**(R)-ketamine:** Preclinical findings have suggested (R)-ketamine has the potential for more potent and longer-lasting antidepressant effects than both ketamine and (S)-ketamine and it appears to have less behavioural side-effects and abuse liability (Chang et al., 2019; Fukumoto et al., 2017; Yang et al., 2015; Zanos et al., 2016). Given initial findings from Vollenweider et al. (1997) in healthy subjects, where (R)-ketamine did not produce psychosis-like symptoms as seen with (S)-ketamine, but instead feelings of relaxation and wellbeing, researchers have now begun to explore the antidepressant potential of (R)-ketamine in humans (Leal et al., 2020).

In the first open-label pilot study of (R)-ketamine, seven subjects with TRD received a single IV infusion of (R)-ketamine at a dose of 0.5 mg/kg over 40 mins (Leal et al., 2020). Mean Montgomery-Åsberg Depression Rating Scale scores dropped significantly from 30.7 at baseline to 10.4 at day 1 after the infusion, with 71% of subjects showing an antidepressant response at day 1 and 57% at day 7. Interestingly, dissociation was nearly absent with minimal haemodynamic effects. However, it should be noted that five out of the seven patients in this study were taking antipsychotic medications (quetiapine (three), risperidone (one), aripiprazole (one)) that might have resulted in lower blood pressure or less dissociation. Naturally, the results of this small open-label study must be interpreted with caution. A clinical trial is underway by *Perception Neuroscience* to further investigate safety and tolerability of differing doses of (R)-ketamine in healthy volunteers before exploring its potential in depression (Universal Trial Number: U1111-1241-1005). In addition, a large trial comparing the efficacy and safety of (R)-ketamine with (S)-ketamine and (R,S)-ketamine in TRD is already underway in China (ChiCTR1800015879).

## Mechanistic considerations

### NMDA receptor antagonism and α-amino-3-hydroxy-5-methyl-4-isoxazolepropionic acid receptor activation

It has been widely acknowledged that the rapid antidepressant effects of ketamine are mediated through blockade of NMDA receptors located on γ-aminobutyric acid (GABA)-ergic inhibitory interneurons (Krystal et al., 2019a). This in turn leads to a disinhibition of pyramidal cells and an acute cortical glutamate surge. Subsequent activation of α-amino-3-hydroxy-5-methyl-4-isoxazolepropionic acid (AMPA) receptors appears to play a key role in the antidepressant effects as demonstrated by preclinical work that has shown pre-treatment with an AMPA receptor antagonist blocks the antidepressant effects of ketamine and its enantiomers in rodent models (Autry et al., 2011; Maeng et al., 2008; Yang et al., 2015).

It has been reported that the metabolism of (R,S)-ketamine to HNK, is required for ketamine’s antidepressant-like effects in rodents (Zanos et al., 2016). Administration of the (R)-enantiomer (2R,6R)-HNK was associated with greater and longer-lasting antidepressant effects than (2S,6S)-HNK and MK-801, a more potent NMDA receptor antagonist. Importantly, (2R,6R)-HNK lacked ketamine-related side-effects and abuse potential in this model. Furthermore, the antidepressant effects were independent of any action on NMDA receptors but instead required AMPA receptor activation (Zanos et al., 2016). Subsequent work has also suggested an important role of presynaptic group II metabotropic glutamate (mGlu2) autoreceptor inhibition in the antidepressant actions of (2R,6R)-HNK (Zanos et al., 2019). Although findings with regards to antidepressant-like effects of (2R,6R)-HNK in rodents have been replicated by other independent laboratories (Fukumoto et al., 2019; Pham et al., 2018), other studies from one laboratory were unable to reproduce this and instead suggest unmetabolised (R)-ketamine itself may be responsible for the antidepressant actions (Shirayama and Hashimoto, 2018; Yamaguchi et al., 2018; Yang et al., 2017a). Clinical trials have not yet examined the utility of (2R,6R)-HNK as a rapid-acting antidepressant. However, clinical studies investigating ketamine metabolite plasma levels as biomarkers have found that higher (2R,6R)-HNK levels were associated with less improvement in depressive symptoms (Farmer et al., 2020; Grunebaum et al., 2019), which is counterintuitive considering preclinical findings. Regardless, (2R,6R)-HNK, with its potential to modulate mGlu2 and AMPA receptor function, remains a promising candidate antidepressant and work to validate this compound for clinical use is ongoing at the United States National Institute for Mental Health (Kraus et al., 2019).

(S)-ketamine is primarily metabolised to (S)-norketamine and it has been shown in an animal model that although the antidepressant actions of the metabolite are similarly potent to the parent compound, (S)-norketamine appeared to lack the associated side effects (Yang et al., 2018a). Importantly, the findings of this study suggest that AMPA receptor activation is not necessary for the antidepressant actions of (S)-nortketamine as AMPA receptor antagonists did not block its antidepressant effects, instead highlighting a role for brain-derived neurotrophic factor (BDNF), tropomyosin kinase B (TrkB) and mechanistic target of rapamycin complex (mTORC) signalling (Yang et al., 2018a). There is, however, some recent clinical evidence that found no relationship between norketamine concentration (neither (S)-norketamine nor (R)-norketamine) and antidepressant response, following administration of (R,S)-ketamine to individuals with TRD (Farmer et al., 2020).

### GABAergic interneuron inhibition

The preferential action of (R,S)-ketamine at GABAergic interneurons is supported by findings that the NMDA receptor antagonist MK-801 initially inhibits the firing of fast-spiking GABAergic interneurons and, at a delayed rate, increases the firing rate of pyramidal neurons (Homayoun and Moghaddam, 2007). Widman et al. (2018) have since demonstrated that perfusion of hippocampal rat brain slices with (R,S)-ketamine enhances the excitability of pyramidal cells indirectly by reducing synaptic GABAergic inhibition, thus causing disinhibition. A recent study found that knockdown of a key NMDA receptor subunit, GluN2B, on GABAergic interneurons resulted in a significant increase (disinhibition) of spontaneous excitatory postsynaptic currents on layer V pyramidal cells in mouse brain slices (Gerhard et al., 2020). Moreover, knockdown of GluN2B on GABAergic interneurons but not pyramidal cells of the medial prefrontal cortex (mPFC) had antidepressant-like effects and occluded or blocked the antidepressant behavioural effects of (R,S)-ketamine. Further supporting this disinhibition hypothesis, administration of negative allosteric modulators of GABA_A_ receptors (GABA-NAMs) exert rapid antidepressant actions similar to (R,S)-ketamine in animal models (Fischell et al., 2015; Zanos et al., 2017), likely through disinhibition of excitatory glutamatergic neurotransmission (Towers et al., 2004). Although GABAergic interneuron inhibition via NMDA receptors appears to serve as an important initial target of (R,S)-ketamine, further work is needed to determine if this mechanism is as relevant for each of ketamine’s enantiomers and metabolites.

### BDNF-TrkB signalling

BDNF and its receptor TrkB have been consistently implicated in the aetiology of depression and mechanism of action of current antidepressants (Duman and Monteggia, 2006; Dwivedi, 2009; Hashimoto et al., 2004). BDNF serves a key a role in processes including neuronal maturation, synapse formation and synaptic plasticity (Park and Poo, 2013). Findings from preclinical work suggest BDNF-TrkB signalling in the hippocampus and prefrontal cortex to be a critical component of antidepressant response to conventional antidepressants (Adachi et al., 2008; Rantamaki et al., 2007; Schmidt and Duman, 2010). The rapid antidepressant-like effects of (R,S)-ketamine have been shown in one preclinical study to depend on the rapid synthesis of BDNF (Autry et al., 2011). In this study, (R,S)-ketamine was shown to rapidly increase TrkB phosphorylation, an indicator of TrkB activation, in the hippocampus, suggesting BDNF-TrkB signalling in this brain region is also involved in the antidepressant response to ketamine. This is in agreement with previous work showing the acute antidepressant effects of (R,S)-ketamine administration are associated with increased BDNF protein levels in the hippocampus (Garcia et al., 2008).

In the study by Autry et al. (2011), the ketamine-mediated suppression of resting NMDA receptor activity was also shown to deactivate eukaryotic elongation factor 2 (eEF2) kinase, resulting in reduced eEF2 phosphorylation and augmentation of BDNF synthesis. Subsequent findings confirmed the importance of this signalling pathway in the antidepressant response to ketamine as eEF2 kinase knockout mice, administered an acute low dose of (R,S)-ketamine did not show an antidepressant response or an increase in BDNF protein expression in the hippocampus (Nosyreva et al., 2013). In addition to the hippocampus, BDNF in the mPFC may also be an important site of action as preclinical work has found that an infusion of a BDNF neutralising antibody into the mPFC abolishes ketamine’s antidepressant-like effects (Lepack et al., 2014). Additional preclinical work demonstrated that (R,S)-ketamine-induced antidepressant effects are associated with upregulation of BDNF and mTORC in the hippocampus and prefrontal cortex, mediated by AMPA receptors (Zhou et al., 2014).

Considering the individual enantiomers, in a chronic social defeat stress and learned helplessness models of depression, a TRkB antagonist was able to block the antidepressant effects of both (S)-ketamine (Yang et al., 2018a) and (R)-ketamine (Yang et al., 2015). Interestingly, (R)-ketamine induced greater effects on reduced dendritic spine density, BDNF-TrkB signalling and synaptogenesis in the prefrontal cortex and hippocampus compared with (S)-ketamine and (R)-ketamine showed a greater potency and longer-lasting antidepressant effect than (S)-ketamine in this model (Yang et al., 2015).

### Mammalian target of rapamycin complex and extracellular signal-regulated kinase

The mammalian target of rapamycin complex 1 (mTORC1) and extracellular signal-regulated kinase (ERK) are key signalling molecules in pathways that regulate protein synthesis with roles in synaptic development and plasticity (Ignacio et al., 2016; Mendoza et al., 2011). The function of mTORC1 and ERK in the antidepressant actions of ketamine and its enantiomers are not completely clear. Work in rodents initially demonstrated that (R,S)-ketamine rapidly activated the mTORC pathway, leading to increased synaptic signalling proteins and synaptic spine density (Li et al., 2010). Furthermore, intracerebroventricular administration of an mTORC1 inhibitor, rapamycin, has been shown to block ketamine-induced synaptogenesis and antidepressant-like effects (Li et al., 2010, 2011). Other work has shown that (R,S)-ketamine administration did not alter levels of phosphorylated mTOR in the hippocampi of control or BDNF-knockout mice, neither were the antidepressant effects of (R,S)-ketamine blocked by intraperitoneally administered rapamycin (Autry et al., 2011). However, this study did report reduced phosphorylation of ribosomal protein s6 kinase in brain tissues, a pharmacodynamic readout of mTORC1 inhibition, following rapamycin administration. One explanation for the failure of rapamycin to block the antidepressant effects of (R,S)-ketamine in the study by Autry et al. (2011) is that the peripheral route of administration may not have achieved sufficient central nervous system exposure compared to studies where intracortical rapamycin administration resulted in adequate mTORC1 inhibition to block (R,S)-ketamine’s antidepressant effects (Li et al., 2010, 2011).

**Figure 3.**
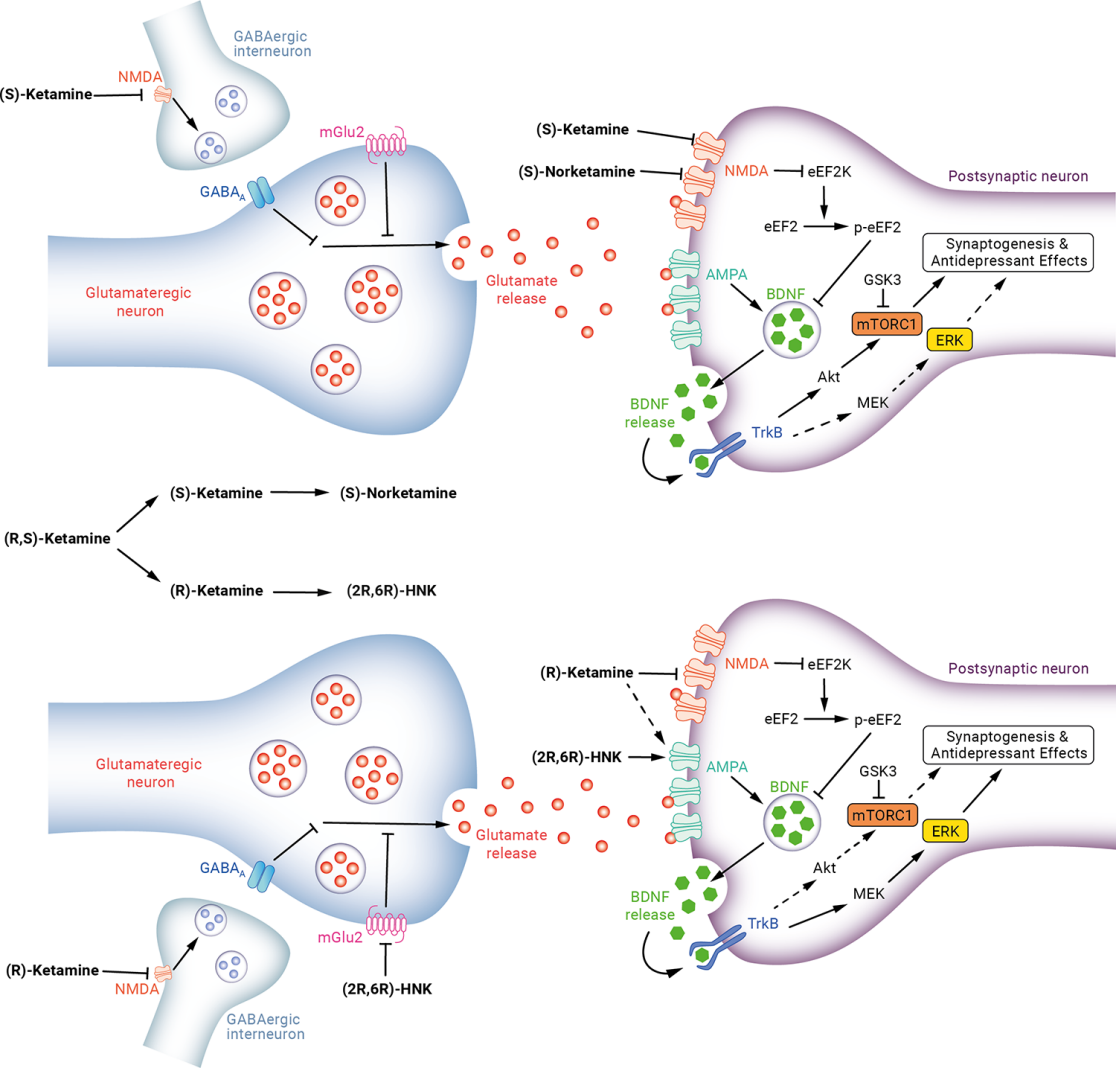
Proposed signalling pathways underlying the antidepressant actions of ketamine enantiomers and metabolites. Top: (S)-ketamine causes glutamate release via disinhibition of γ-aminobutyric acid (GABA) interneurons. Resulting glutamate surge stimulates α-amino-3-hydroxy-5-methyl-4-isoxazolepropionic acid (AMPA) receptors leading to release of brain-derived neurotrophic factor (BDNF) with resulting activation of tropomyosin kinase B (TrkB)-Akt-mammalian target of rapamycin complex 1 (mTORC1) signalling. This leads to increased synthesis of proteins required for synaptogenesis. (S)-ketamine and (S)-norketamine suppress resting N-Methyl-D-Aspartate (NMDA) receptor activity, deactivating eukaryotic elongation factor 2 (eEF2) kinase, resulting in reduced eEF2 phosphorylation, augmentation of BDNF synthesis and TrkB-mTORC1 activation. Bottom: (R)-ketamine causes glutamate release via disinhibition of GABA interneurons with activation of AMPA receptors and BDNF release but there may be an alternative pathway by which (R)-ketamine stimulates AMPA receptor transmission that still needs to be elucidated. (R)-ketamine may cause preferential activation of TrkB-MEK-ERK signalling pathway leading to synaptogenesis. (2R,6R)-HNK directly activates AMPA receptors and inhibition of group II metabotropic glutamate (mGlu2) receptors may also be involved in this metabolite’s antidepressant actions.

Further preclinical work demonstrated that the antidepressant effects of (S)-ketamine but not (R)-ketamine were blocked by mTORC1 inhibition and that (S)-ketamine, but not (R)-ketamine, significantly attenuated decreased phosphorylation of mTOR in the prefrontal cortex of mice in a chronic social defeat stress model (Yang et al., 2018b). This same study showed that pre-treatment with an ERK inhibitor blocked the antidepressant effects of (R)-ketamine but not (S)-ketamine and, furthermore, (R)-ketamine but not (S)-ketamine significantly attenuated the reduced phosphorylation of ERK in the prefrontal cortex and hippocampi of susceptible mice using the same model (Yang et al., 2018b). It is interesting to note that the antidepressant effects of (S)-norketamine, (S)-ketamine’s predominant metabolite, have also been shown to be blocked by the mTORC1 inhibitor rapamycin (Yang et al., 2018a). Taken together this suggests that mTORC1 has a role in antidepressant effects of (S)-ketamine but less so for (R)-ketamine and that ERK activation could instead mediate the antidepressant effects of (R)-ketamine (Figure 3).

In a recent randomised double-blind cross-over study, Abdallah et al. (2020) explored whether pre-treatment with rapamycin could attenuate the rapid antidepressant effects of (R,S)-ketamine in individuals with MDD. Surprisingly, rapamycin did not alter the acute antidepressant effects of (R,S)-ketamine but instead prolonged the antidepressant effects (Abdallah et al., 2020). Two weeks after ketamine administration there were significantly higher response and remission rates following rapamycin + ketamine compared to placebo + ketamine. The authors hypothesised that the failure to block ketamine’s effects by rapamycin may have been due to the dosage used and peripheral route of administration, mirroring the findings from preclinical work that also utilised peripheral rapamycin administration (Autry et al., 2011). A key difference between this human work and the preclinical work, however, is follow-up time. None of the ketamine + rapamycin animal studies discussed could have discovered findings as shown in the study by Abdallah et al. (2020) as antidepressant effects were not followed up for long enough (Autry et al., 2011; Li et al., 2010, 2011). Abdallah et al. (2020) also hypothesised that rapamycin may extend the antidepressant effects of ketamine via an mTORC1-dependent anti-inflammatory mechanism (Thomson et al., 2009), protecting newly made synapses from inflammatory processes that cause synaptic elimination and undermine the antidepressant effects of ketamine, or by enhancing autophagy (a crucial mTORC1 regulated process involved in normal cellular plasticity, which involves degrading and recycling toxic or dysfunctional cellular components) (Abdallah et al., 2020). Alternatively, it is possible to speculate rapamycin may have mTORC1-independent effects that contribute to the antidepressant effects of ketamine. For example, rapamycin may attenuate an unacknowledged homeostatic mechanism that normally contributes to relapse. A final speculative consideration is whether rapamycin may promote longer-lasting antidepressant effects of (R)-ketamine via increased ERK signalling as low concentrations of rapamycin have been shown to increase Akt and ERK activation in vitro through an mTORC1-dependent mechanism (Chen et al., 2010). Although interesting, these preliminary findings should be interpreted with caution and replication in future studies is needed, before back translation to animal studies (Abdallah and Krystal, 2020), alongside work to determine any differential effects of rapamycin on each of ketamine’s enantiomers.

### Glycogen synthase kinase 3

Activation of mTORC1 signalling has been linked to phosphorylation (deactivation) of glycogen synthase kinase-3 (GSK-3) and inhibition of GSK-3 has been shown to be necessary for the rapid antidepressant-like effects of (R,S)-ketamine in mice (Beurel et al., 2011). Furthermore, administration of (R,S)-ketamine in combination with lithium, a non-selective GSK-3 inhibitor, resulted in rapid activation of the mTORC1 signalling pathway, increased inhibitory phosphorylation of GSK-3, increased synaptic spine density and potentiated antidepressant-like responses in rodents (Liu et al., 2013). Ketamine-induced inhibition of GSK-3 has also been linked to AMPA receptor upregulation and stabilisation at the cell surface. In a preclinical study it was demonstrated that (R,S)-ketamine-induced inhibition of GSK-3 resulted in reduced phosphorylation of post-synaptic density-95 (which regulates AMPA receptor trafficking), diminishing the internalisation of AMPA GluA1 subunits (Beurel et al., 2016). This could ultimately allow for augmented signalling through AMPA receptors following ketamine treatment.

In clinical work, testing the GSK-3 inhibition hypothesis, lithium continuation therapy showed no benefit over placebo at 2 weeks following the cessation of four (R,S)-ketamine infusions in individuals with TRD (Costi et al., 2019). In patients with treatment-resistant bipolar depression, maintained on either therapeutic-dose lithium or valproate before receiving (R,S)-ketamine versus placebo, a significant improvement in depressive symptoms was seen in both mood stabiliser groups, and although ketamine’s antidepressant effect size relative to placebo was larger for lithium (*d*=2.27) than valproate (*d*=0.79), there was no significant difference observed between these two agents (Xu et al., 2015). Furthermore, neither serum lithium nor valproate levels correlated with ketamine’s antidepressant efficacy.

Although some evidence highlights GSK-3 as an important regulatory target for ketamine’s antidepressant effects, clinical studies have not yet confirmed preclinical findings. Further evaluation of the role of GSK-3 in the antidepressant effects of ketamine’s individual enantiomers and metabolites is still required.

### Translocation of G_s_ alpha subunit from lipid rafts

An increase in intracellular cyclic adenosine monophosphate (cAMP) that acts to upregulate neurotrophic factors and increase synaptogenesis has been associated with conventional antidepressant action (Dwivedi and Pandey, 2008; Gass and Riva, 2007). Gα_s_ is a subunit of the G protein G_S_ that stimulates the generation of cAMP by activating adenylyl cyclase. Localisation of Gα_s_ within lipid raft microdomains in the plasma membrane acts to regulate cellular signalling, and indeed production of cAMP is diminished when Gα_s_ is localised to lipid raft microdomains (Allen et al., 2009). A number of preclinical studies have demonstrated increases in cAMP through translocation of Gα_s_ from lipid raft domains into non-raft regions, augmenting interaction between Gα_s_ and adenylyl cyclase, following administration of various classes of antidepressants (Czysz et al., 2015; Toki et al., 1999; Zhang and Rasenick, 2010).

Wray et al. (2019) have since reported that (R,S)-ketamine administration to C6 glioma cells led to immediate translocation of Gα_s_ from lipid raft domains to non-raft domains and an increase in cAMP, followed by an increase in BDNF expression after 24 hours. The (R,S)-ketamine induced increase in cAMP was found to persist after knocking out the NMDA receptor indicating an NMDA receptor-independent mechanism. Further, administration of the ketamine metabolite (2R,6R)-HNK also resulted in redistribution of Gα_s_ from lipid rafts and an increase cAMP production. These findings suggest the translocation of Gα_s_ from lipid rafts is a reliable hallmark of antidepressant action; however, further research is needed to examine to what degree this mechanism contributes to the antidepressant effect of the individual enantiomers of ketamine.

### Monoaminergic systems

Several studies suggest that 5-hydroxytryptamine (5-HT) signalling plays a role in the antidepressant effects of ketamine. Preclinical work has demonstrated that the antidepressant-like action of (R,S)-ketamine is blocked by pre-treatment with a 5-HT-depleting agent (Fukumoto et al., 2014, 2016; Gigliucci et al., 2013). (R,S)-ketamine has been found to inhibit serotonin transporter (SERT) function in vitro (Zhao and Sun, 2008) and a positron emission tomography (PET) study in conscious monkeys further reported that subanaesthetic (R,S)-ketamine selectively enhanced serotonergic transmission by inhibition of SERT activity (Yamamoto et al., 2013). Alongside SERT inhibition, increased mPFC 5-HT release via AMPA receptor stimulation in the dorsal raphe nucleus may be involved in the antidepressant effects of (R,S)-ketamine (Chaki and Fukumoto, 2019; Pham et al., 2017). Moreover, it has been demonstrated that an mPFC infusion of a 5-HT_1a_ receptor antagonist blocks the antidepressant-like effects of (R,S)-ketamine in mice and attenuates ketamine-induced increases in phosphorylation of Akt (Fukumoto et al., 2018). (R,S)-ketamine antidepressant effects were mimicked by intra-mPFC, but not systemic, administration of a 5-HT_1a_ receptor agonist and both the antidepressant effects of ketamine and the 5-HT_1a_ receptor agonist were blocked by the mTORC1 inhibitor rapamycin (Fukumoto et al., 2018). Finally, in a recent study, infusion of a selective 5-HT_1A_ receptor agonist into the mPFC produced ketamine-like rapid synaptic and antidepressant-like behavioural responses in a rodent model that were blocked by co-infusion of an AMPA receptor antagonist (Fukumoto et al., 2020). Taken together, it appears another route via which (R,S)-ketamine may cause its antidepressant effects is through 5-HT_1A_ receptor activation in the mPFC, by AMPA receptor-dependent 5-HT release, with downstream convergence on signalling mechanisms. These include the Akt/mTORC1 pathway but may also include ERK signalling, which is also activated by direct 5HT_1A_ receptor stimulation (Buritova et al., 2009; Newman-Tancredi et al., 2009).

Considering the individual enantiomers, an in vivo microdialysis study has shown that both (R)- and (S)-ketamine acutely increase 5-HT release in the prefrontal cortex, with (R)-ketamine causing a greater increase than (S)-ketamine (Ago et al., 2019). Although the (S)-ketamine-induced 5-HT release was attenuated by an AMPA receptor antagonist, (R)-ketamine-induced 5-HT release was not affected by AMPA receptor blockade. Although preclinical work has demonstrated that 5-HT depletion abolishes the antidepressant-like actions of (S)-ketamine in a genetic model of depression (du Jardin et al., 2016), other work has shown that 5-HT depletion does not alter the antidepressant effects of (R)-ketamine in a chronic social defeat stress model (Zhang et al., 2018). This suggests that 5-HT may not play as major a role in antidepressant effects of (R)-ketamine. The reason for these differences is not entirely clear and further work is needed to explore the role of 5-HT in the effects of ketamine and its enantiomers.

The dopamine system has also been implicated in depression and the antidepressant effects of ketamine; however, the mechanism underlying the action of ketamine or its enantiomers on this system has not been fully established. Acute subanaesthetic (R,S)-ketamine administration is associated with significantly increased dopamine levels in the cortex, striatum and nucleus accumbens in rodents (Kokkinou et al., 2018) and there is also in vivo PET imaging evidence that (R,S)-ketamine and (S)-ketamine administration leads to increased striatal dopamine release in humans as indexed by D_2_/D_3_ receptor tracer binding (Breier et al., 1998; Smith et al., 1998; Vollenweider et al., 2000). A further PET study found that IV (S)-ketamine administration, but not (R)-ketamine, led to a significant reduction of binding availability of dopamine D_2_/D_3_ receptor in the monkey striatum and suggests that unlike (R)-ketamine, (S)-ketamine can cause dopamine release in the striatum that may contribute to the psychotomimetic/dissociative side effects in humans (Hashimoto et al., 2017). Other groups found that (R,S)-ketamine-induced reductions of D_2_/D_3_ binding in humans only occurred in combination with amphetamine, suggesting ketamine may enhance the sensitivity of the dopamine system but not lead to direct dopamine release (Aalto et al., 2002, 2005; Kegeles et al., 2002).

In a study examining the effects of (R,S)-ketamine and metabolites on evoked striatal dopamine release and dopamine receptors, (R,S)-ketamine did not alter the magnitude or kinetics of electrical stimulation-evoked dopamine release in the nucleus accumbens of anesthetised mice and neither ketamine’s enantiomers nor its metabolites had affinity for dopamine receptors or the dopamine transporter (Can et al., 2016). This suggests the side effects and antidepressant actions of ketamine (or its metabolites) may not be associated with direct effects on mesolimbic dopaminergic neurotransmission. An alternative hypothesis is that ketamine produces indirect effects through NMDA receptor antagonism on GABAergic interneurons, resulting in disinhibition of glutamatergic projections on to dopamine neurons in the midbrain, an increase in glutamate release, subsequent activation of dopaminergic neurons and increased dopamine levels in targets such as the striatum and cortex (Stone et al., 2007).

Additional research findings indicate that dopamine D_1_ receptor activity in the mPFC are necessary for the rapid antidepressant actions of (R,S)-ketamine using optogenetic stimulation in a mouse model (Hare et al., 2019). Another potential convergence from a signal transduction perspective may involve NMDA and D_1_ receptor-dependent induction of mTORC/ERK and inactivation of eEF2 kinase resulting in increased protein synthesis (David et al., 2020). Further work found that pre-treatment with a dopamine D_1_ receptor antagonist did not block the antidepressant effects of (R)-ketamine in a chronic social defeat stress model (Chang et al., 2020). Furthermore, although (S)-ketamine has been shown to cause a robust increase in dopamine release compared with (R)-ketamine, the antidepressant-like effects were more potent and longer acting following (R)-ketamine administration in a mouse model (Ago et al., 2019). Taken together, these findings suggest that activation of the dopamine system may be required for the antidepressant actions of (S)- but not (R)-ketamine.

### Inhibition of lateral habenula bursting

Increasing lines of preclinical and clinical evidence highlight a major role for the lateral habenula, an anti-reward centre, in the pathophysiology of depression. It is suggested that abnormal increases in neuronal activity in this region signal downregulation of brainstem dopaminergic and serotonergic firing, resulting in depressive symptomatology including anhedonia, helplessness and excessive focus on negative experiences (Gold and Kadriu, 2019).

Recent work from Yang et al. (2018c) found that blockade of NMDA receptor-dependent bursting activity in the lateral habenula mediated the antidepressant actions of (R,S)-ketamine in rodent models of depression. It was demonstrated that lateral habenula bursting required both NMDA receptors and low-voltage-sensitive T-type calcium channels. Furthermore, administration of T-type calcium channel inhibitors (ethosuximide and mibefradil) caused rapid antidepressant-like effects in both the forced swim test and sucrose preference test (Yang et al., 2018c). In contrast, preclinical work utilising a chronic social defeat stress model failed to demonstrate antidepressant effects of ethosuximide whereas (R)-ketamine showed rapid and long-lasting antidepressant actions in this model (Tian et al., 2018). Furthermore, a recent double-blind RCT in medication-free patients with depression found no significant reductions in depression and anxiety scores following treatment with ethosuximide, suggesting T-type calcium channel inhibitors are unlikely to exert ketamine-like robust antidepressant actions (Zhang et al., 2020a).

It should be noted that inhibition of lateral habenula bursting as a mechanism of antidepressant action has only been assessed acutely at 1 hour post (R,S)-ketamine infusion (Yang et al., 2018c). Whether this mechanism is active later during (R,S)-ketamine’s antidepressant effects (>24 hours) or indeed if there are differential effects of (S)- and (R)-ketamine on lateral habenula bursting remains unknown.

### Opioid receptor system

Ketamine interacts with mu, kappa and, to a lesser extent, delta-opioid receptors (Ki = 42.1, 28.1, and 272 mΜ, respectively) (Hirota et al., 1999; Zanos et al., 2018). The affinity of (S)-ketamine for the mu and kappa opioid receptors is two to fourfold that of (R)-ketamine (Hirota et al., 1999; Hustveit et al., 1995). Recent work demonstrated that pre-treatment with naltrexone, an opioid receptor antagonist, significantly blocked the antidepressant and anti-suicidal effects of (R,S)-ketamine in TRD, suggesting that opioid system activation was necessary for the rapid-acting antidepressant and antisuicidal effects of (R,S)-ketamine (Williams et al., 2018, 2019). There are a number of important limitations to these studies including the small sample size (only 12 participants completing both naltrexone and placebo pre-treatment conditions and only seven of the 12 meeting response criteria during the ketamine plus placebo condition), lack of a placebo control arm for the (R,S)-ketamine infusion (i.e., naltrexone + IV saline and placebo + IV saline) and finally that participants may have experienced a noxious, nocebo type of response to the naltrexone + ketamine treatment, which influenced subsequent depression ratings (Mathew and Rivas-Grajales, 2019). Other work has demonstrated that naltrexone pre-treatment did not affect the antidepressant effects of (R,S)-ketamine in depressed individuals with alcohol use disorder (Yoon et al., 2019) and an earlier study in healthy individuals found that the behavioural effects of an antidepressant dose of ketamine were potentiated by pre-treatment with naltrexone (Krystal et al., 2006). Finally, in patients with TRD, concurrent use of buprenorphine and methadone (high affinity mu opioid receptor agonists) was not associated with reductions in antidepressant efficacy of (R,S)-ketamine (Marton et al., 2019).

In rodent models of depression (chronic social defeat stress and inflammation induced), it was shown that naltrexone pre-treatment did not block the antidepressant effects of (R,S)-ketamine (Zhang and Hashimoto, 2019). However, in a subsequent preclinical study, it was shown that opioid antagonists abolish the ability of (R,S)-ketamine to reduce depression-like behavioural and lateral habenula cellular hyperactivity (Klein et al., 2020). The authors suggested the opioid system is ‘necessary but not sufficient’ for the antidepressant actions of (R,S)-ketamine in rodents as activation by morphine, a mu-opioid agonist, at a dose high enough to induce a hedonic response, did not mimic the rapid antidepressant-like effects of ketamine or reduce lateral habenula neuronal activity (Klein et al., 2020). The authors argued that in their studies of lateral habenula cellular activity, (R,S)-ketamine did not appear to act as a mu-opioid agonist but that some mu-opioid receptor activity was necessary for NMDA receptor antagonism. In brain regions, including the habenula, NMDA receptors and opioid receptors display colocalisation (Rodriguez-Munoz et al., 2012) and NMDA receptor activation can be modulated by actions of opioid receptors (Kow et al., 2002; Martin et al., 1997). Taken together, this suggests a potential interaction that may be explained by direct ‘crosstalk’ between the glutamatergic and the opioid receptor systems (Chartoff and Connery, 2014), or by convergence at downstream signalling pathways.

**Figure 4.**
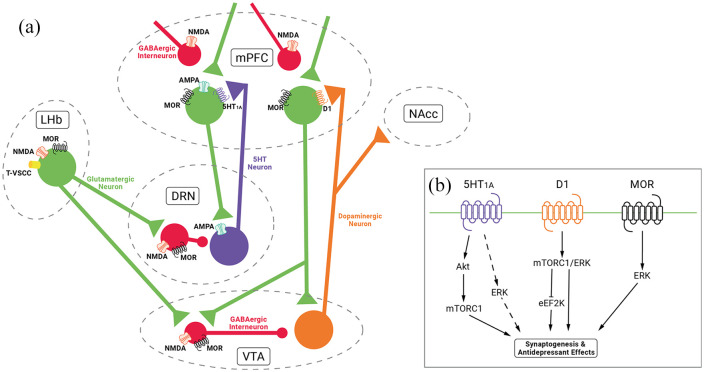
Hypothesised monoamine and opioid mechanisms and potential convergences with signalling pathways implicated in the antidepressant actions of ketamine. (a) (R,S)-ketamine inhibits lateral habenula (LHb) bursting via actions on N-Methyl-D-Aspartate (NMDA)/low voltage sensitive t-type channels (T-VSCC)/mu-opioid receptors (MOR). This results in disinhibition of monoamine release via γ-aminobutyric acid (GABA)-ergic interneurons in the dorsal raphe nucleus (DRN) and ventral tegmental area (VTA) to projections including the medial prefrontal cortex (mPFC) and nucleus accumbens (NAcc). Action of (R,S)-ketamine on NMDA/MOR on GABAergic interneurons in the DRN and VTA may be a further mechanism of disinhibition of 5-HT and dopamine release. 5-HT release in mPFC may also occur via α-amino-3-hydroxy-5-methyl-4-isoxazolepropionic acid (AMPA) receptor stimulation in DRN for (R,S)-ketamine and (S)-ketamine but might not be as relevant for (R)-ketamine. (b) Stimulation of postsynaptic 5-HT_1A_ receptors via 5-HT in mPFC results in activation of Akt/mammalian target of rapamycin complex 1 (mTORC1) and potentially ERK signalling. Stimulation of postsynaptic D1 receptor via dopamine may result in activation of mTORC1/ERK and inactivation of eukaryotic elongation factor 2 (eEF2) kinase. Postsynaptic MOR activation may also potentiate the ERK signalling pathway.

There are several potential convergences between opioid signalling and other mechanisms implicated in the antidepressant action of ketamine. For example, administration of endogenous opioids has been shown to upregulate BDNF expression in the frontal cortex, hippocampus and amygdala (Zhang et al., 2006). Moreover, these effects were reversed by naltrexone administration. Acute mu-opioid receptor activation has been shown to result in rapid activation of ERK signalling (Bian et al., 2015; Zheng et al., 2008) and acute treatment with ketamine enhances the levels of opioid-induced ERK phosphorylation in cells that endogenously express mu-opioid receptors (Gupta et al., 2011). Further, administration of an opioid receptor antagonist, naloxone, has been shown to inhibit mu-opioid-induced ERK activation in a dose-dependent manner in C6 glioma cell lines (Gutstein et al., 1997). Finally, there are a number of functional interactions between opioid receptors and monoaminergic systems relevant to mood control (Lutz and Kieffer, 2013). Specifically, activation of mu-opioid receptors expressed in the dorsal raphe nucleus and ventral tegmental area, via GABAergic interneurons, disinhibit 5-HT (Fadda et al., 2005; Tao and Auerbach, 2002) and dopamine neurons (Le Merrer et al., 2009) with projections including the prefrontal cortex and nucleus accumbens (Lutz and Kieffer, 2013) (Figure 4).

The role of the opioid system in the antidepressant effect of ketamine remains controversial and a topic of debate (Amiaz, 2019; Heifets et al., 2019; Krystal et al., 2019b; Sanacora, 2019). Further work with rigorous trial design and parallel mechanistic studies are required to understand the function of the ketamine-opioid receptor interaction and subsequent signalling cascades in the antidepressant effect of each of ketamine and its individual enantiomers.

## Conclusion

**Figure 5.**
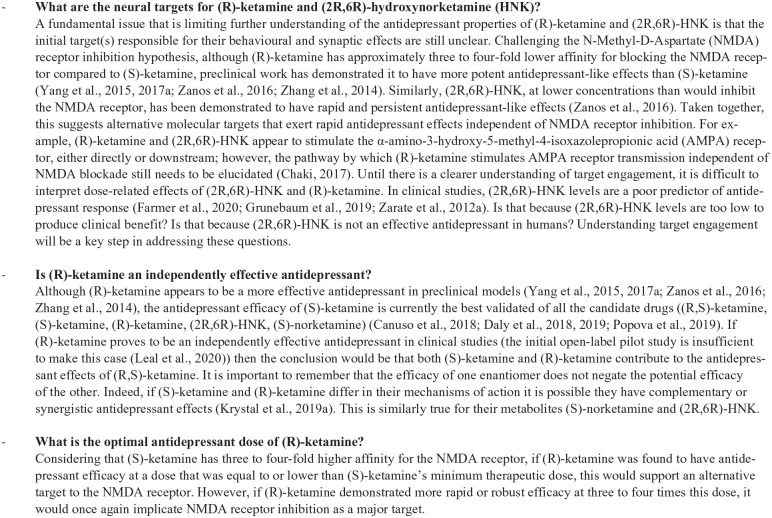
Outstanding questions.

The discovery of the rapid antidepressant effects of (R,S)-ketamine, including in treatment-resistant patients, has appropriately been hailed ‘the most important discovery in half a century’ in depression research (Duman and Aghajanian, 2012). Through the drug development and clinical trials process, the (S)-ketamine nasal spray, *Spravato*^TM^, has been approved in both the United States and Europe, although some concerns remain regarding efficacy and side effects. The first pilot study of (R)-ketamine in TRD has demonstrated encouraging results and, considering preclinical findings, it appears (R)-ketamine may have a more favourable safety profile than (S)-ketamine. Accumulating preclinical evidence also suggests (R)-ketamine to have more potent and longer-lasting antidepressant effects than both (R,S)-ketamine and (S)-ketamine. As studies of (R)-ketamine progress through Phase I and Phase II, results from direct comparison studies of the safety and efficacy of (R)-ketamine and (S)-ketamine in TRD will be crucial. Other key outstanding questions are outlined in Figure 5.

Although NMDA receptor inhibition and subsequent AMPA receptor activation have a role in the antidepressant effects of ketamine, further mechanistic work is building a more nuanced understanding of the distinct molecular and cellular mechanisms of ketamine, its enantiomers and metabolites, including BDNF-TrkB, mTORC1 and ERK signalling. Although there may be a role for monoaminergic and opioid receptor systems in the antidepressant effects or detrimental side effects of ketamine, further work examining the effects of each of the component enantiomers on these systems is required. All the while, new pieces of the ketamine puzzle are being discovered and other potential future directions of enquiry include examining the role of the transforming growth factor β1 system (Zhang et al., 2020b) and the brain-gut-microbiome axis (Huang et al., 2019; Yang et al., 2017b) in the antidepressant effects of ketamine and its enantiomers.

As we further our understanding of the similarities and differences in the signalling pathways associated with (S)-ketamine, (R)-ketamine and their metabolites, we should bear in mind potential complementary or synergistic antidepressant effects that might arise via distinct mechanisms. A deeper understanding of the precise molecular and cellular mechanisms underlying the antidepressant effects and negative side effects of (R,S)-ketamine, (S)-ketamine and (R)-ketamine will be invaluable as we seek to develop future rapid-acting antidepressants with favourable safety profiles, alongside treatment strategies to maintain adequate response.
